# Engineered Vascular Beds Provide Key Signals to Pancreatic Hormone-Producing Cells

**DOI:** 10.1371/journal.pone.0040741

**Published:** 2012-07-12

**Authors:** Keren Kaufman-Francis, Jacob Koffler, Noa Weinberg, Yuval Dor, Shulamit Levenberg

**Affiliations:** 1 Department of Biomedical Engineering, Technion - Israel Institute of Technology, Haifa, Israel; 2 Biotechnology Interdisciplinary Unit, Technion - Israel Institute of Technology, Haifa, Israel; 3 Department of Cellular Biochemistry and Human Genetics, Faculty of Medicine, Hebrew University of Jerusalem, Jerusalem, Israel; Johns Hopkins University, United States of America

## Abstract

The mechanisms underlying early islet graft failure are not entirely clear, but are thought to involve ischemic injury due to delayed vascularization. We hypothesize that blood vessels play an active role in cell-cell communications supporting islet survival and engraftment. To test this hypothesis and to uncouple endothelial cell (EC)-generated signaling stimuli from their nutritional and gas exchange functions, we developed three dimensional (3D) endothelial vessel networks in engineered pancreatic tissues prepared from islets, fibroblasts and ECs. The tri-culture setup, seeded on highly porous biocompatible polymeric scaffolds closely mimics the natural anatomical context of pancreatic vasculature. Enhanced islet survival correlating with formation of functional tube-like endothelial vessels was demonstrated. Addition of foreskin fibroblasts to islet-endothelial cultures promoted tube-like structure formation, which further supported islet survival as well as insulin secretion. Gene expression profiles of EC growth factors, extracellular matrix (ECM), morphogenes and differentiation markers were significantly different in 2D versus 3D culture systems and were further modified upon addition of fibroblasts. Implantation of prevascularized islets into diabetic mice promoted survival, integration and function of the engrafted engineered tissue, supporting the suggested role of ECs in islet survival. These findings present potential strategies for preparation of transplantable islets with increased survival prospects.

## Introduction

Islet transplantations have been attracting increasing attention in recent years, as they carry new hope for successful restoration of glycemic control in Type 1 diabetes patients [1]. However, only moderate success has been achieved due to donor organ shortage and low islet allograft survival rates, increasing the number of islets required per procedure. One of the most likely reasons for the poor success rates relates to the avascular nature of islets grafts, following their collagenase-based purification and free transplantation [2], [3]. As a result, transplanted islets are wholly dependent on host vascularization machinery for reestablishment of the vascular system, which is indispensable to prolonged survival [4]. During the time interval required for such revascularization, islets are highly susceptible to ischemic injury and increased mortality [5], [6].

Recent studies have shown that the endothelium produces a large number of growth factors, cytokines and other molecules which solicit paracrine effects unrelated to their role in blood [7]. More specifically, a number of studies have described the role of blood vessels in promoting embryonic development of the pancreas, even in the absence of blood flow [8], [9], [10], [11], suggesting an integral role of islet-associated endothelial cells (ECs) in islet survival, proliferation and differentiation. Thus, we hypothesize that the non-nutritional EC-stimulated signals may be paramount to *in vitro* culturing of islets for the purpose of boosting early graft infusion survival prospects. In this manner, ECs may be critical to enhancing success rates of islet-based therapy.

The present work aims to elucidate the nature of EC-islet interdependence, its reliance on the tissue microenvironment and its role in islet graft survival. To this end, advanced tissue-engineering techniques were applied to develop a novel three-dimentional (3D) multi-culture model comprised of pancreatic islets, ECs and fibroblasts in efforts to reconstruct vascularized pancreatic tissue *ex vivo*. The experimental setup allows for disengagement of blood vessels from their nutritional function [12], [13]. In addition, through such a culture system design, both ECs, which organize into tubular structures, and pancreatic tissue retain their natural architecture, allowing for a close look at the physiological interactions transpiring between the two cell types [8], [14], [15]. Such cell culture conditions substantiated the hypothesized essential role of 3D vascular beds in enhancing both islet survival and physiological functioning. Implantation of 3D vascularized islet constructs into diabetic mice led to improved engraftment profiles and to significant reductions in blood glucose levels. These findings support the notion that re-establishment of functional 3D microvasculature can significantly boost islet survival *in vitro* and post-implantation prospects *in vivo*.

## Results

### Engineering 3D Vascularized Islets

**Figure 1:**
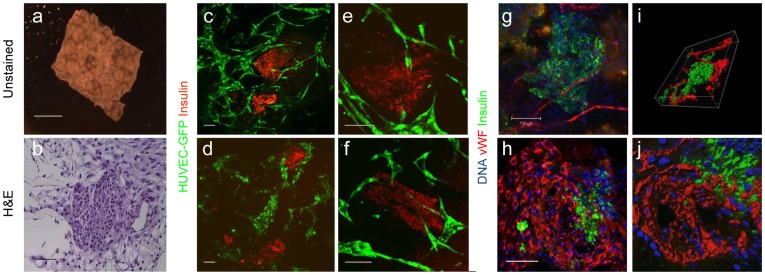
*In vitro* vascularization of islet multicultures. 10-day-old multi-cellular cultures of mouse pancreatic islets, human ECs and HFFs, grown on 3D PLLA/PLGA polymer scaffolds were analyzed. (**a**) Whole scaffold, bright field photo; scale bar- 2mm. (**b**) A paraffin-embedded section stained with H&E; scale bar-200 µm. (**c–f**) Stereomicroscope confocal images of engineered vascularized pancreatic islet scaffolds stained for insulin (red) and HUVEC-GFP (green). (**g–h**) Laser scanning confocal image of a cell-embedded scaffold stained for insulin (green), vWF (red) and nuclear content (blue); scale bar-100 µm. (**i–j**) 3D reconstruction of images collected in (g–h) using the Imaris software.

In efforts to mimic the natural anatomical context of the pancreatic vascular network *ex vivo*, tissue engineering techniques were used to developed a novel 3D multicellular model, in which mouse islets and human umbilical vein endothelial cells (HUVECs) were cultured together with human foreskin fibroblast (HFF) mesenchymal cells [16], [17], on highly porous and biodegradable PLLA/PLGA scaffolds (Fig. 1a,b). Confocal imaging of 10-day old constructs revealed self-arrangement of endothelial cells into interconnected, branched vessel-like structures, situated in close proximity to the islets, and bearing close resemblance to the 3D lumen of blood vessels (Fig. 1c–f). Furthermore, 10-day old tri-culture-embedded scaffolds demonstrated well-organized, von Willebrand factor (vWF)-expressing tubes encircling the pancreatic islets (Fig. 1g–j).

### The 3-dimensional Organization of the Engineered Vascular Network Plays a Key Role in Pancreatic Islet Survival Ex vivo

**Figure 2:**
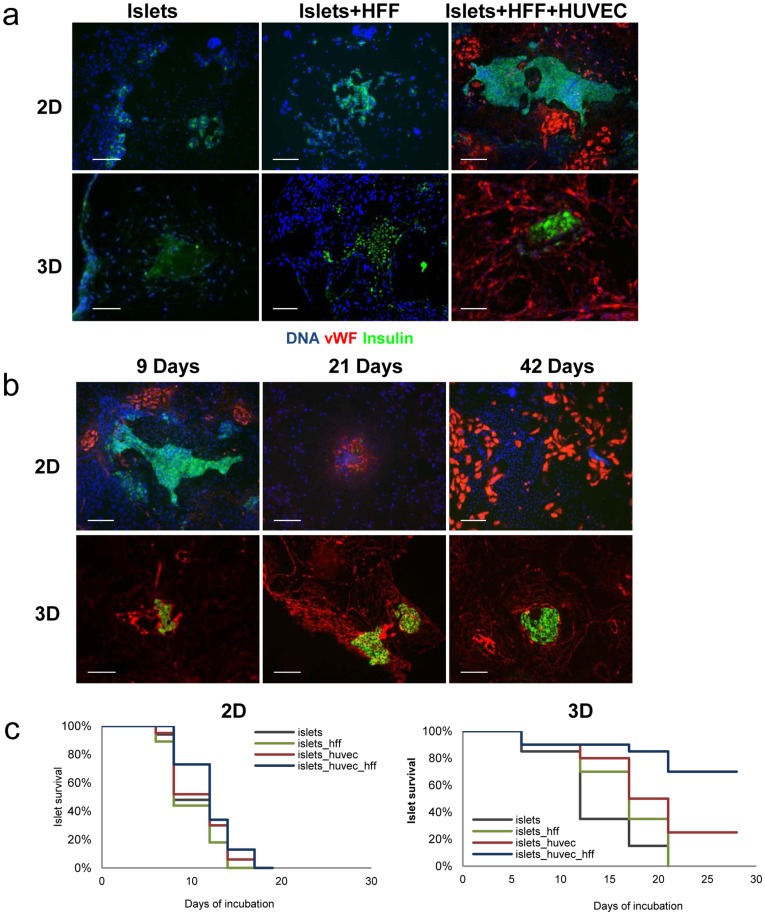
*In vitro* vascularization of engineered pancreatic tissue: 2D versus 3D growth environments. 20 isolated mouse islets seeded alone, with HFF or with HFF and HUVEC were either grown on 3D polymer scaffolds or on 2D ECM-coated plates for up to 28 days. Scaffolds and plates were triple-stained with anti-vWF (red), anti-insulin (green) antibodies and DAPI (blue). (**a**) 10-day old, immunostained sections; scale bar-200 µm. (**b**) Staining of 9-, 21- and 42-day-old sections. Scale bar-200 µm (**c**) Islet survival in 2D and 3D cultures was determined by quantification of the number of viable islets under light microscope using trypan blue, insulin and DAPI staining for the 2D-plated islets and immunofluorescently via insulin- and DAPI-stained islets for 3D scaffolds.

An islet survival assay was then set up to allow for quantification of the contribution of engineered vascular microenvironments to islet viability *ex vivo*. Islet-embedded scaffolds, cultured in the presence or absence of ECs and/or HFFs, were fixed and embedded in formalin at different points along a four-week incubation period. Double vWF and insulin cross-section labeling confirmed the central role of 3D lumen-forming ECs in morphological preservation and survival of islets, grown in the absence of blood flow. Islets tri-cultured with ECs and HFFs on 3D scaffolds remained intact and expressed insulin even after 28 days in culture; 80% were still viable by the end of the experiment (Fig. 2a–c). On day 10, islet morphology was preserved in >80% of the islets co-cultured with ECs and 90% of the islets cultured with ECs and HFF (Fig. 2a, c). 3D islet-EC co-cultures persisted for 28 days as well, but with a 75% islet mortality rate (p<0.05), (Fig. 2c). In contrast, most of the islets seeded on EC-free scaffolds were disintegrated; 100% cell death was observed within 21 days, where 60% had already dissolved within ten days of seeding. A 10–15% loss of islets was detected in all 10 day-old islet cultures grown on 3D scaffolds and may have occurred during the seeding procedure (Fig. 2c).

On the basis of previous reports describing the critical role of 3D tissue organization on cellular interactions and signaling [18], we anticipated provision of environmental cues, lacking in 2D co-culture settings, by this unique 3D setup. To further examine the impact of variant spatial organization of ECs on preservation of islet morphology and physiology, islet behavior on 2D ECM-coated plates was compared to that of those cultured on scaffolds. Samples were incubated for up to 4 weeks and observed every other day. Dramatic differences in both EC organization and islet viability were recorded between 2D and 3D culture systems. While round EC clusters and rings were observed in close interaction with islets grown in 2D co- and tri-cultures, configuration of 3D vessels was not supported (Fig. 2a, b). In contrast, 3D multicellular cultures enabled ECs to form elongated, interconnecting tubes yielding vascular network-like structures, which surrounded the islets (Fig. 2a). In addition, islets grown in 3D multicellular cultures tripled the average lifetime compared to isles grown alone in the 3D culture, and doubled the average life time compared to islet cultured on scaffolds with HFFs only (Fig. 2c). The islets cultured in the 3D multicellular culture preserved their morphology even after 42 days of culture (Fig. 2b). While no significant role was attributed to HFFs when cultured alone with islets in 2D settings, in the 3D settings, on days 12–17 they induced a 30% improvement in islet survival (p<0.05) compared to islets cultured alone (Fig. 2c). On the same days, addition of ECs to the 3D islet culture resulted in a 40% improvement in islet survival compared to the survival of islets cultured alone (Fig. 2c). Taken together, these data stress the importance of endothelial 3D vessel-like structures in islet survival.

**Figure 3:**
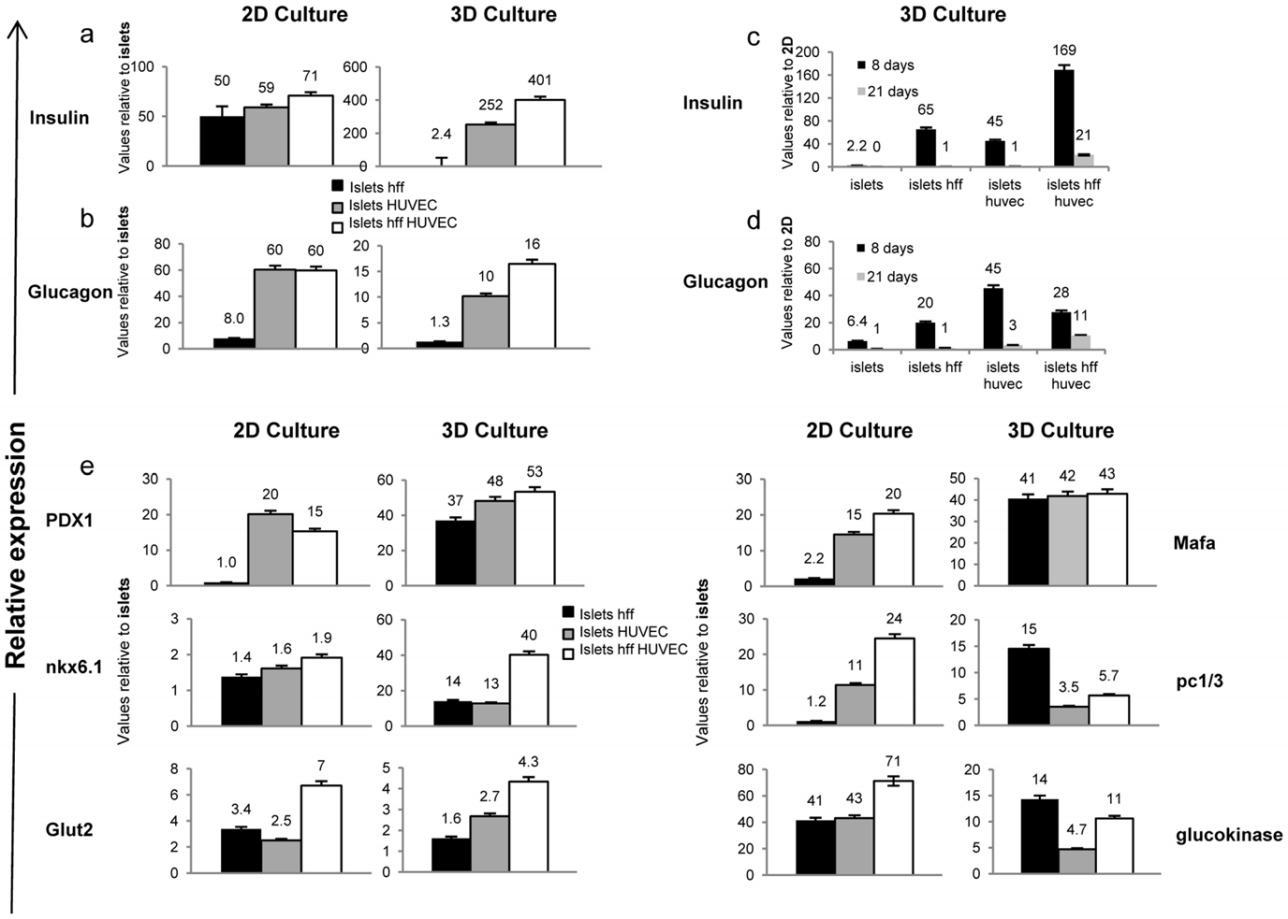
Addition of endothelial cells to islet cultures promotes islet function *in vitro.* Real-Time PCR analyses of (**a**) insulin and (**b**) glucagon were performed on islets cultured in 2D or 3D microenvironments for 8 days, in the absence or presence of HFF and/or HUVEC. Values were normalized to mouse 18s and are presented as the recorded levels versus those of free islets cultured under the same conditions. Differential expression analyses of (**c**) insulin and (**d**) glucagon were performed by comparing their levels in 3D versus 2D islet culture on days 8 and 21. Values were normalized to mouse 18s RNA and are presented as the ratio of levels expressed in 3D versus 2D islet culture environments. (**e**) Real-Time PCR analyses of the mature islet genes *PDX1, NKX6.1, GLUT2, MAFA, PC1/3* and glucokinase were performed for islets cultured in 2D and 3D microenvironments, in the absence or presence of HFF and/or HUVEC. Values were normalized to mouse 18s and are presented as the ratio of the recorded levels versus those of free islets cultured under the same conditions.

**Figure 4:**
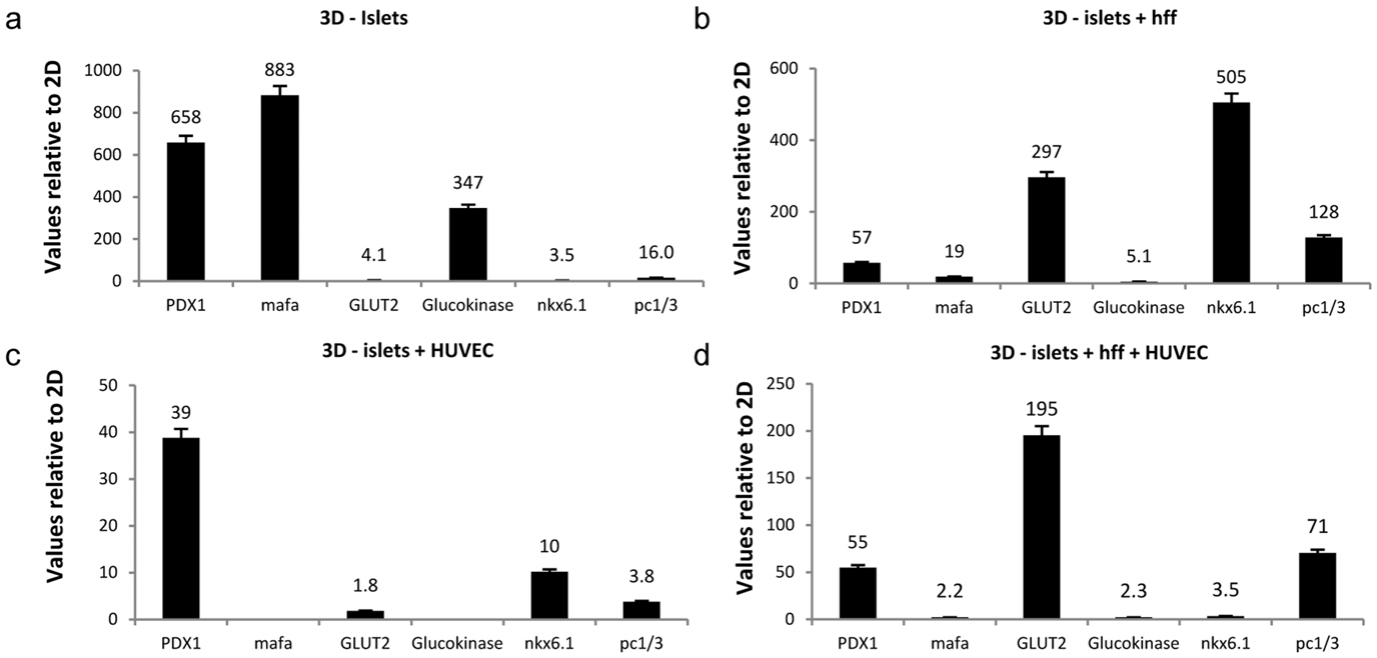
Expression profiles of EC morphogenesis-related genes. Quantitative real-time PCR analyses, using human specific primers were performed on islet-EC settings following their culture (**a**) with HFF in a 3D co-culture system or (**b**) with HFF in a 2D co-culture system. (**c**) Relative gene expression between 3D/2D multicellular culture systems. (**d**) Relative gene expression between 3D/2D co-culture systems. Values were normalized to human GAPDH.

Quantitative RT-PCR analysis performed on 3D scaffolds and 2D ECM-coated plates loaded with pancreatic islets alone, islets co-cultured with HFF, islets co-cultured with ECs or islets tri-cultured with fibroblasts and ECs further strengthened the above findings. Addition of HFF or ECs to the 2D culture led to a ≥50-fold increase in insulin expression, when compared to islets cultured alone (Fig. 3a) for 8 days. A similar increase in glucagon expression was observed for islet-EC 2D cultures, when compared to islets cultured alone (Fig. 3b). In contrast, 3D scaffold islet cultures demonstrated a 252-fold increase in insulin expression in the presence of ECs and a 401-fold increase when grown in the tri-culture system (Fig. 3a). Significant upregulation of glucagon expression was also noted for the 3D tri-culture (Fig. 3b). When comparing 2D to 3D cultures at 8 days post-seeding, a 169-fold increase in insulin and 28-fold increase in glucagon expression were observed in 3D tri-cultures versus their 2D equivalents (Fig. 3c, d). After 21 days, the difference in gene expression between 3D and 2D cultures decreased considerably (Fig. 3c,d), but still remained higher in 3D environments. Differential expression analysis of an extensive list of islet marker genes including *PDX1*, glucokinase, *GLUT2, MAFA, NKX6.1* and *PCSK1* demonstrated elevated mRNA levels in both 2D and 3D settings, upon inclusion of HFF, ECs or both, in islet cultures (Fig. 3e). However, mRNA levels detected in 3D cultures were consistently higher than those recorded for 2D cultures (Fig. 4).

### Differential Gene Expression in Endothelial Cells and Islets Grown in 2D Versus 3D Pancreatic Culture Systems

In efforts to further characterize EC gene expression patterns associated with improved islet survival and function, EC gene expression profiles in both 2D and 3D islet cultures were analyzed by Taqman low density array. Expression levels of genes encoding differentiation markers, morphogenes, adhesion molecules, growth factors, ECM components and transcription factors were considerably modified in 3D islet-EC-HFF tri-cultures, when compared to 3D islet-EC co-cultures (Fig. S1). Expression of growth factor receptors such as KDR, EPHA1 and EPHA7 was dramatically reduced upon addition of HFF to EC-islet cultures, in both 3D and 2D settings (Fig. S1a,b). Downregulation of proliferation- and cytoskeleton-related genes was also observed, suggesting fibroblast-driven stimulation of EC differentiation rather than proliferation. In parallel, elevations in gene expression profiles of differentiation markers, morphogenes, apoptosis regulators, growth factors, ECM components, transcription factors and cytoskeletal proteins in 3D multicellular culture systems were recorded in 3D, but not in 2D multicellular systems (Fig. S1c). A dramatic 400% rise in RGS-5 G-protein regulator expression was measured in 3D tri-cultures versus their 2D counterparts, possibly reflecting differentiation into mural cells and vascular maturation developing in the 3D tri-culture system [19] (Fig. S1a). In, contrast, comparison between 2D and 3D islet-EC systems (Fig. S1d) demonstrated a >30% downregulation of the *FN1, FGF2* and *VEGF* genes, often associated with pericyte-mediated vessel stabilization [20]. Overall, the minimal changes observed in gene expression profiles upon addition of fibroblasts to 2D islet-EC cultures, further highlight their impact in the 3D setup (Fig. S1B).

**Figure 5:**
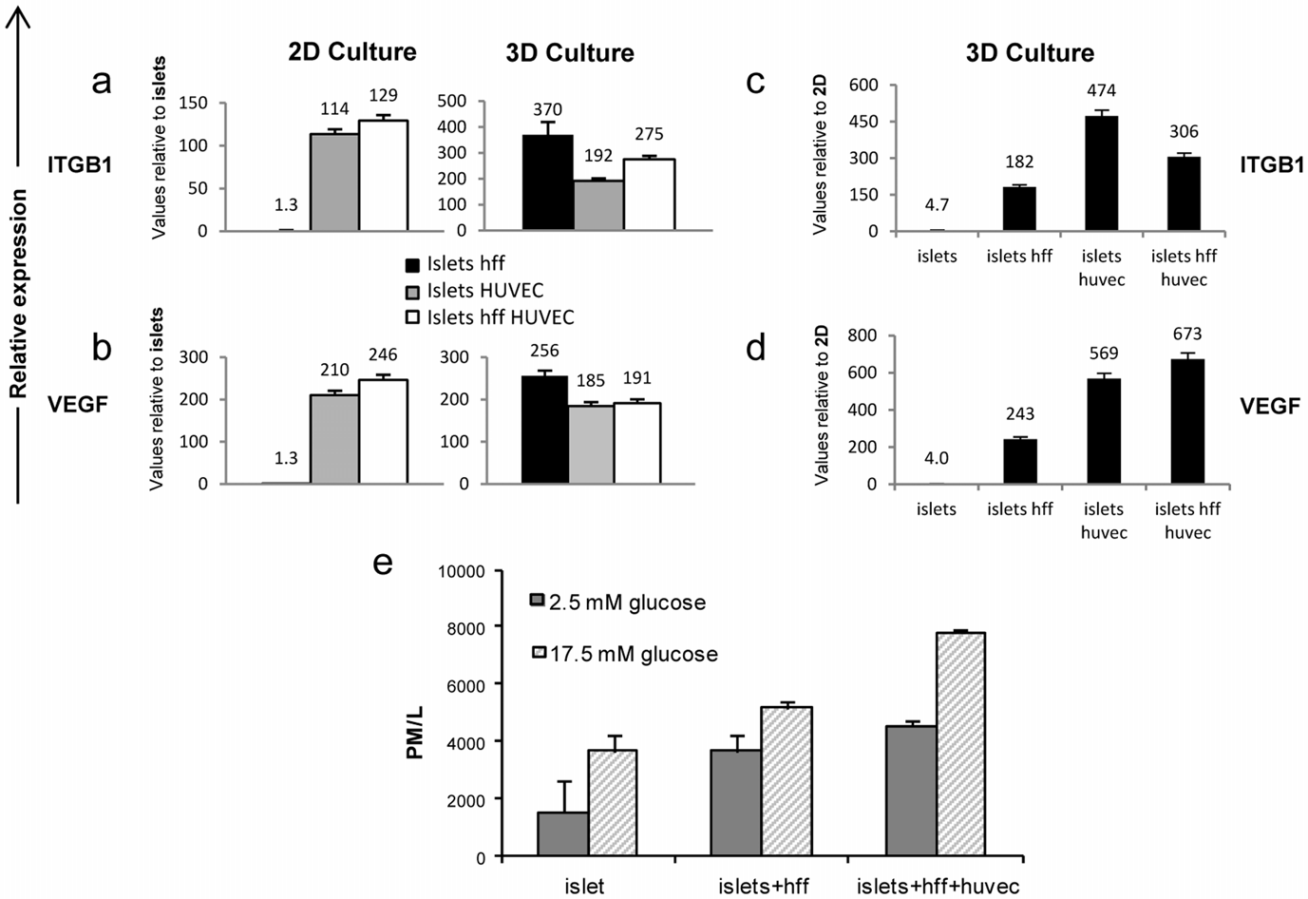
Endothelial cells promote upregulation of ECM-associated genes in islet cultures. Quantitative real-time PCR analyses of the ECM-associated (**a**) *ITGB1* and (**b**) *VEGF* genes were performed on islets cultured in 2D or 3D microenvironments in the absence or presence of HFF and/or HUVEC for 8 days**.** Values were normalized to mouse 18s and are presented as the fold increase in recorded expression levels versus those of free islets cultured under the same conditions. Expression analyses of (**c**) *ITGB1*and (**d**) VEGF-A were performed by comparing 3D to 2D settings of various cell cultures. Values are presented as the fold increase in expression levels of 2D versus 3D cultures. (**e**) c-peptide concentrations in KRB media were measured from 8-day-old cultures of 3D scaffolds seeded with islets, islets + HFF or islets + HFF + HUVEC after challenge with low glucose doses (2.5 mM) and high glucose doses (17.5 mM).

Focus on gene expression of EC basement membrane proteins as a possible source of the endothelial signaling on adjacent islets, indicated upregulation of EC laminins and collagen IV genes in 3D tri-cultures (Fig. S1a,c), as well as elevation of islet β1 integrin subunit (*ITGB1*) in both 2D and 3D cultures upon addition of ECs or ECs and HFF to islet cultures (Fig. 5a). A 192-fold and a 275-fold increase in islet *ITGB1* expression was documented when islets were cultured with ECs or with ECs and HFF, respectively on 3D scaffolds in comparison to islets cultured alone. An even greater increase in *ITGBI* expression was observed in the 3D islet-HFF co-cultures, compared to islets cultured alone (Fig. 5a). Islet-derived VEGF-A, an additional factor considered to be involved in islet-endothelial interactions [21] was dramatically elevated in islet cultures under the influence of ECs or ECs and HFF, in both 2D and 3D environments, when compared to islets cultured alone (Fig. 5b). However, comparative analyses of islets cultured in 3D versus 2D environments demonstrated consistently higher *ITGB1* and *VEGF-A* expression in all 3D culture groups (Fig. 5 c,d), where the most significant elevation (673-fold) was measured for *VEGF-A* in 3D tri-cultures, when compared to the 2D tri-cultures (Fig. 5d).

### 3-dimensional Engineered Vascular Networks Play a Key Role in Insulin Secretion

The 3D experimental platform was then used to test the role of vascular beds in preservation of islet insulin release in response to glucose challenge. Twenty islets were cultured alone, with fibroblasts only or tri-cultured with ECs and fibroblasts on 3D scaffolds for a period of 8 days. C-peptide secretion was measured after low (2.5 mM) and high (17.5 mM) glucose challenge. High glucose doses led to an increase in c-peptide secretion in the presence or absence of ECs, when compared to low-glucose conditions (p<0.001) (Fig. 5e).

However, significant differences between the groups were already noticed after the low glucose challenge as c-peptide levels of islets co-cultured with fibroblasts or tri-cultured with ECs and fibroblasts were 2.45 and 3.04 higher than those of islets cultured alone (p<0.001). In addition, mean c-pepide secretion levels of islet tri-culture were 23% higher than the c-peptide level of isles co-cultured with fibroblasts.

Following the high glucose challenge, induced c-peptide levels in islet tri-cultured with fibroblasts and ECs were 1.13 higher compared to c-peptide levels in stimulated islets cultured alone (p<0.001). Addition of ECs to the culture system increased the c-peptide secretion levels by 51% compared to the c-peptide secretion level of islets co-cultured with just fibroblasts (p<0.001).

### In-vivo Implantation of 3D Engineered Vascularized Pancreatic Tissue

**Figure 6:**
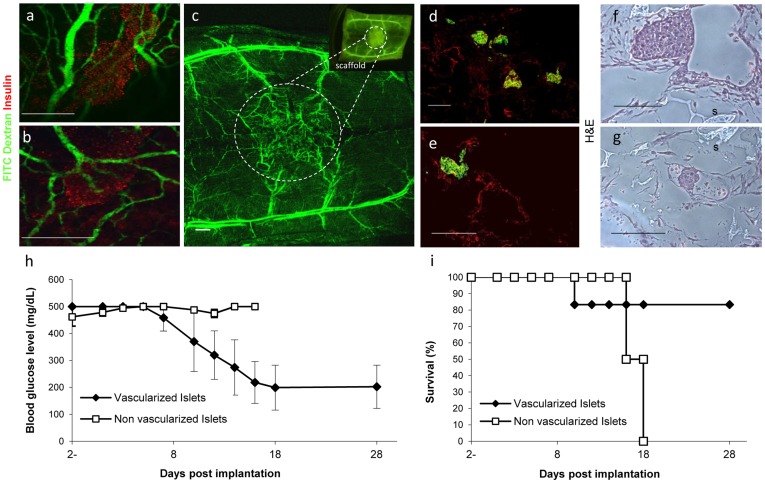
*In vivo* biofunctioning of 3D vascularized islet constructs. 5-day old vascularized or non-vascularized 3D islet constructs were implanted into the peritoneal subcutaneous area of streptozotocin-induced diabetic mice. (**a, b**) 3D pre-vascularized constructs were removed 2 weeks after implantation, sectioned and immunostained for insulin (green) and vWF (red) to identify human ECs. Scale bar-200 µm. (**c, d**) Histological examination of H&E-stained, implanted vascularized islet constructs, retrieved 1 week following restoration of blood glucose levels (200 mg/dl) in each animal. S = scaffold. Scale bar-200 µm. (**e, f**) Vascularized islet implants were retrieved from mice 14 days post-implantation and following injection of FITC-dextran to the tail vein. Samples were then immunofluorescently stained with anti-insulin antibodies (red) and viewed with a laser scanning confocal stereomicroscope (Leica). Scale bar-100 µm. (**g**) Functional, FITC-dextran-perfused vessels in the engineered vascularized islet implant, viewed with a laser scanning confocal stereomicroscope on day 14 post-implantation. Scale bar-200 µm. (**h**) Blood glucose levels and (**i**) Survival of streptozotocin-induced diabetic mice after implantation of vascularized or non-vascularized islet-bearing scaffolds (50 islets per scaffold) implanted after a 5-day culturing period (vascularized implant, n = 6, non- vascularized, n = 4).

In order to evaluate the capacity of the vessel networks created *in vitro* to promote further vascularization, integration and insulin expression *in vivo,* grafts were implanted into mice and assessed for viability and physiological behavior. A single 3D vascularized (n = 6) or non-vascularized (n = 4) pancreatic construct consisting of 50 islets, was implanted into the subcutaneous space of the peritoneal area of a streptozotocin-induced diabetes mellitus (DM) nude mouse. Blood vessel formation in the area of the implant, as well as graft survival and reduction in blood glucose levels were analyzed (Fig. 6). Injection of FITC-dextran into the mouse tail vein demonstrated functional, interconnected blood vessel networks within the implanted scaffold area, suggesting implant-host vasculature integration and functional implant vessels (Fig. 6a–c). H&E or insulin/vWF immunofluorescence staining of extracted prevascularized islet scaffolds highlighted viable islets surrounded by vessel-like vWF-expressing structures (Fig. 6d–g). In addition, reduced blood glucose levels accompanied with improved animal survival rates were only seen in mice bearing vascularized islet implants (Fig. 6h,i p<0.05), indicating construct integration and islet functionality. Taken together, implantation of prevascularized constructs containing 50 islets enhanced engraftment success rates and reduced blood glucose levels in DM mice.

## Discussion

In this study, biodegradable polymeric scaffolds were utilized to establish a 3D tri-culture system of human ECs, human foreskin fibroblasts and isolated mouse islets, in an attempt to mimic the natural anatomical context of pancreatic vasculature (Fig. 1). This multicellular culture system was expected to support and stabilize pancreatic tissue prepared *ex vivo* and to ultimately enhance islet cell viability and survival *in vivo*. The use of human- versus mouse-specific primers (Table S1) and/or antibodies, facilitated differentiation of gene expression patterns and histological analysis in this mixed mouse/human tissue model.

The unique 3D culture design enabled self-assembly of ECs to lumen-containing vascular structures and upregulation of a number of known angiogenesis regulators. In parallel, distinct differences in islet survival rates were noted between islets cultured in the presence versus the absence of ECs (Fig. 2c), indicating their critical role in islet support, and substantiating the significant impact of their geometric configuration in pancreatic islet fate. From a functional angle, islets embedded in 3D vascularized scaffolds demonstrated enriched levels of insulin and glucagon gene expression, as well as other islet marker genes, when compared to the 2D vascular environment (Fig. 3). In parallel, an increase in c-peptide secretion was observed in these 3D tri-cultures. Of note, ECs from other sources, including MS1 (ATCC), were tested and gave similar results.

Overall, the modified gene expression profiles reported here suggest that ECs undergo substantial changes during 3D morphogenesis, especially with regard to ECM components and growth factors associated with tissue microenvironments, and presumably involved in generation and support of the lumen-like structures (Fig. S1c). In addition, upregulation of the EPHA1 and RGS5 tyrosine kinases was recorded, and is suggested to have played a role in initiating the molecular control required for the prevascularization process. The islet β1 integrin subunit, which forms laminin receptors upon association with various α-integrin subunits and is required for the β cell response to EC laminins [22], was dramatically upregulated, especially in 3D tri-cultures (Fig. 5a,c). Similarly, islet VEGF-A, associated with ECM arrangement [23], was upregulated in response to EC signals in the 3D model (Fig. 5b).Experiments performed by the Lammert group, have demonstrated that during later embryonic development, delaminated β-cells aggregate to form islets expressing high levels of VEGF-A. In turn, these islets attract VEGFR2-expressing ECs, which form a vascular network within the islets [9], [10]. These findings suggest that ECM synthesis occurs during the 3D vascularization process, and serves as a factor central to the survival and behavior of cultured islets.

Addition of HFF to the islet-EC 3D culture system correlated with self-organization of vascular networks, which highly influenced islet behavior and improved islet survival *in vitro* as well as graft infusion. We have previously shown that inclusion of fibroblasts in cultures designed to engineer tissue structures, supports fibroblast recruitment and differentiation into pericytes and smooth muscle (SM) cells [17], which later form elongated endothelial tubular structures promoting tissue vascularization and maturation [16]. In parallel, islet-ECs cultured with HFF in a 3D setting demonstrated dramatic changes in EC and HFF gene expression profiles, most prominently in ECM and growth factor-related genes (Fig. S1a); changes of this nature were not observed in 2D cultures of identical cell mixtures (Fig. S1b). The EPHA1 and EPHA7 ephrin receptors were dramatically downregulated in all cases where HFFs were included in islet-EC cultures (Fig. S1a,b), suggesting that alternative regulatory mechanisms such as angiopietin receptors or VEGF receptors are involved in the endothelial-mesenchymal interaction. In parallel, HFFs significantly influenced islet viability and c-peptide secretion in 3D cultures, as well as islet gene expression in both 2D and 3D setups, even in the absence of ECs (Fig. 3, Fig. 5 and Fig. S1). These data support earlier reports of fibroblast-generated FGF signaling as being directly responsible for stimulating mouse pancreatic β-cell protein activity, such as those of PC1/3 and glucokinase [24]. In summary, the HFF-driven boost in islet viability reflects a potential role of mesenchymal cells in islet survival and function.

C-peptide has been reported to play an active role in preventing endothelial dysfunction under high glucose conditions by binding to specific cell membrane G-protein receptors of both fibroblasts and ECs [25]. In light of the results observed in the present study, we hypothesize that under high glucose concentrations part of the secreted c-peptide molecules are bound to the fibroblast and EC receptors, specifically inhibiting NF-κB and other cellular factors involved in high glucose-induced vascular dysfunction.

Finally, blood glucose levels and histology results coupled with FITC-dextran fluorescence (Fig. 6), demonstrated vasculature-related promotion of implant integration *in vivo*, which led to increased blood perfusion at the implant site and improved engraftment. As hypothesized, prevascularization of implants supported graft-host anastomosis, and boosted neovascularization and angiogenesis processes at the implant site. Moreover, reduction in blood glucose levels was observed within 6 days and mouse survival rates were significantly lengthened.

Taken together, these findings stress the importance of 3D organization, as well as the role of endothelial vessel-like structures in maintaining islet morphology and survival both *in vitro* and *in vivo*. Moreover, these findings support the notion that the islet vascular bed provides key survival signals to pancreatic hormone-producing cells even in the absence of blood flow. The reported data further encourage our long-term goal to design a defined synthetic microenvironment that will reduce the number of islets required for successful islet transplantations.

## Materials and Methods

### Ethics Statement

Experimental procedures and animal care were performed in accordance with the National Research Council’s Guide for the Care and Use of Laboratory Animals. All protocols were reviewed and approved by the Animal Care and Use Committee of the Technion.

### Engineering Three-dimensional Scaffolds

3D scaffolds were prepared using a salt leaching technique [12], blending poly(lactide-co-glycolide) (PLGA; MW = 25,000; Boehringer-Ingelheim) and poly-L-lactic acid (PLLA; MW = 300,000; Polysciences) in a 1∶1 ratio, yielding a final pore size range of 300–600 µm and 93% porosity [26]. These pore sizes were chosen to facilitate seeding and growth of both islets and ECs. Sponges were cut into rectangular pieces of ∼5×4×1 mm^3^ and sterilized in 70% ethanol before use.

### Cell Culture

Pancreatic islets were isolated from 14–18 week old mouse pancreases by injecting collagenase P (Roche) to the bile duct, followed by digestion at 37°C for 15 min. Handpicked islets were cultured in islet culture medium (CMRL-1066, Biological Industries, Beit-Haemek, Israel) supplemented with 10% fetal bovine serum (HyClone, Erembodegem-Aalst, Belgium), 1% penicillin-streptomycin solution (Invitrogen-Gibco,Paisley,USA), 2 mM L-glutamine (Invitrogen-Gibco) and 25 mM HEPES (Invitrogen-Gibco).

Human umbilical vein endothelial cells (HUVEC, Lonza) and HUVEC-GFP (passage 4–6, Angio-Proteomie) were cultured in endothelial cell medium EGM-2® supplemented with 2% FBS and endothelial cell growth medium BulletKit® (Cambrex Bio Science Walkersville, Inc). Human foreskin fibroblast cells (neonatal normal human dermal fibroblast, Lonza) (HFF) were cultured in Dulbecco’s Modified Eagle’s Medium (DMEM, Gibco) supplemented with 10% FBS (Hyclone), 1% nonessential amino acids (NEAA), and 0.2% β-mercaptoethanol (Sigma–Aldrich).

### 3D Islet Cultures

Co-cultures of EC and pancreatic islets were prepared by suspending 5×10^4^ HUVEC or HUVEC-GFP per islet in 15 µl of a 1∶1 mixture of growth factor-reduced Matrigel (BD Biosciences, NJ, USA) and islet culture medium. For tri-cultures, 3×10^4^ HFF were added to the islet-HUVEC co-cultures described above. Cell mixtures of ∼1–2×10^6^ cells were seeded per PLLA/PLGA scaffold. Cell-embedded scaffolds were cultured in 50% endothelial medium and 50% islet culture medium for 1–6 weeks on an orbital shaker, to allow for maximal medium perfusion and to prevent scaffold attachment to the plate.

### 2D Islet Cultures

EC and pancreatic islet co-cultures or tri-culture systems, prepared as described above, were cultured on ECM-coated plates (Novamed, Jerusalem, Israel) in the same medium used for cell-embedded scaffold culturing.

### Islet Survival Assay

Twenty isolated mouse islets were seeded on 3D scaffolds or ECM-coated plates alone, with HFF, with ECs and with ECs and HFF and incubated for 28 days. Quantification of viable islets grown on 2D ECM-coated plates or 3D scaffolds was performed every other day using either trypan blue (for 2D cultures) or by fixating and staining specimens with anti-insulin antibody (for 2D and 3D culture) (Dako, Glostrup, Denmark). For trypan blue staining, growth medium was replaced with PBS containing trypan blue (1∶1 ratio) and cells were observed under the light microscope immediately thereafter. Islets stained with blue were considered nonviable, while islets stained in green were considered viable. In addition, islets stained in green but that had completely lost their typical morphological structure, were also considered nonviable. Islets seeded on 3D scaffolds were observed and counted by means of insulin staining.

### Immunofluorescence Analysis

2D cultures were fixed and permeabilized with 3.2% paraformaldehyde (PFA) +0.5% Triton (5 min, room temperature (RT)), followed by a 25 min fixation in 3.2% PFA. 3D scaffold samples were fixed in 4% PFA (6 hours, RT) and embedded in paraffin. Serial sections were prepared and stained with hematoxylin and eosin (H&E) for tissue organization and vessel formation analysis. 2D and 3D EC-islet samples were labeled with polyclonal rabbit anti-vWF (1∶200) and guinea-pig anti-insulin (1∶75) (Dako, Glostrup, Denmark) antibodies. After extensive washing (3×5 min), sections were incubated with Cy3-donkey anti-rabbit and/or AlexaFluor goat anti-rabbit IgG (Molecular Probes,Oregon, USA), and Cy2-donkey anti-guinea-pig IgG (Jackson ImmunoResearch, PA, USA) antibodies, together with 4′,6-diamidino-2-phenylindole (DAPI) (Sigma). Sections were viewed under a Zeiss Axiovert 200 M microscope. Whole-mount 3D scaffolds were fixated using 4% PFA, permeabilized with 0.2% Triton X-100 and labeled as described above. Stained scaffolds were viewed and images were constructed using laser scanning confocal microscopy (LSM 510 Meta laser scanning confocal system, Zeiss, Germany).

### Gene Expression

RNA was isolated from both 2D and 3D 8-day-old culture systems using Qiagen RNeasy Micro Kits (Qiagen Inc.,Valencia, CA), and RNA yields were determined by spectrophotometry (model ND1000; NanoDrop Technologies, Rockland, DE). cDNA was prepared from total RNA using random hexamer primers (GE Healthcare, Buckinghamshire, UK) and reverse transcriptase (PrimeScript; Takara Bio Inc., Shiga, Japan). Gene expression was detected by Real-Time PCR (Prism 7900HT Sequence Detection System, Applied Biosystems, CA,USA). Each cDNA sample (118 ng) was loaded on to a custom-designed TaqMan Low-Density Array (96 TaqMan® Gene Expression assays preconfigured in a 384-well format, Part no. 437816, microfluidic cards; Applied Biosystems). Expression levels of target genes were normalized to GAPDH (ID Hs99999905_m1; Assay-on-Demand; ABI) for human ECs and 18S RNA (ID Hs99999901_s1; Assay-on-Demand; ABI) for mouse isolated islets. All procedures were performed at The Center for Genomic Technologies at the Institute of Life Sciences in the Hebrew University of Jerusalem, Israel, according to the manufacturer’s instructions. Samples were run in duplicates, and gene expression levels were analyzed using SDS2.3 software (Applied Biosystems).

### Glucose-challenge Studies

3D islet 8-28-day-old cultures were incubated in KRB media, under each of the following conditions: (1) low (2.5 mM) glucose for 30 or 60 minutes, (2) high (17.5 mM) glucose concentrations for 30, 60 or 90 minutes or (3) high (17.5 mM) glucose plus 100 mM 3-isobutyl-1-methylxanthine (IBMX) (Sigma). The supernatant media were collected and c-peptide levels were measured by radioimmunoassay (Linco Research, St. Charles, MO, USA), according to the manufacturer’s protocol.

### Mouse Model for Implantation of Engineered Vasculo-pancreatic Structures

All mice were anesthetized with ketamine and xylazine (127.5 and 4.5 mg/kg, respectively) before performance of any surgical procedures described herein. 3D vascularized or non-vascularized constructs (50 islets per scaffold) were implanted into the subcutaneous space of the peritoneal area of 8-week-old streptozotocin-induced (STZ; Sigma Chemical Co., St. Louis, MO) DM nude mice. Blood glucose (BG) levels were measured every 3 days with a glucose oxidase-based monitor (MediSense Precision Plus, Abbott Laboratories, Doncaster, Victoria, Australia). Mice with non-fasting BG>400 mg/dl were defined as diabetic and were included in the study. Non-STZ mice was checked and was found to be 110–152,mg/dl.

### Host-implant Vessel Anastomosis

Neovessel formation and implant vessel perfusion were assayed two to eight weeks after implantation, by injecting 200 µl (10 mg/ml) FITC-dextran (Sigma Chemical Co., St. Louis, MO) into the mouse tail vein. After an incision through the ventral skin, animals were placed under a fluorescence stereo-microscope for observation. Mice were sacrificed at the end of the experiment and implants were retrieved for immunohistological analysis. Retrieved implants were whole-mount immunofluorescently stained with DAPI and anti-insulin antibody, as described above, and later observed and photographed using laser scanning confocal microscopy (LSM 510 Meta laser scanning confocal system, Zeiss).

### Statistical Analysis

Statistical analysis was performed using JMP 8.0 statistical software (SAS Institute, Inc., Cary, NC). Blood glucose levels in response to the different treatments were analyzed by Dunnett’s test. The therapeutic effect was further verified using the Fit model to test the effect of the different treatments along the experimental period. *In vitro* and *in vivo* survival experiments were analyzed using the Cox-Mantel Rank test.

## Supporting Information

Figure S1 **Expression profiles of EC morphogenesis-related genes.** Quantitative real-time PCR analyses, using human specific primers were performed on islet-EC settings following 8 days of culture (a) with HFF in a 3D multicellular culture system relative to islet-EC cultured without HFF in a 3D co-culture (3D tri-culture vs. 3D co-culture) (b) with HFF in a 2D multicellular-culture relative to islet-EC cultured without HFF in a 2D co-culture system (2D tri-culture vs. 2D co-culture). (c) With HFF in a 3D multicellular culture relative to islet-EC cultured with HFF in a 2D multicellular culture system (3D tri-culture vs. 2D tri-culture). (d) Without HFF in a 3D co-culture relative to islet-EC cultured without HFF in a 2D co- culture system (3D co-culture vs. 2D co-culture). Values were normalized to human GAPDH.
(TIF)

Table S1 **List of primers used for gene expression analysis.**
(DOCX)
