# Supplementary figures and images for: Engineered Vascular Beds Provide Key Signals to Pancreatic Hormone-Producing Cells
Source: PLoS One. 2012 Jul 12;7(7):e40741. doi: 10.1371/journal.pone.0040741 (PMC3395696; doi:10.1371/journal.pone.0040741)

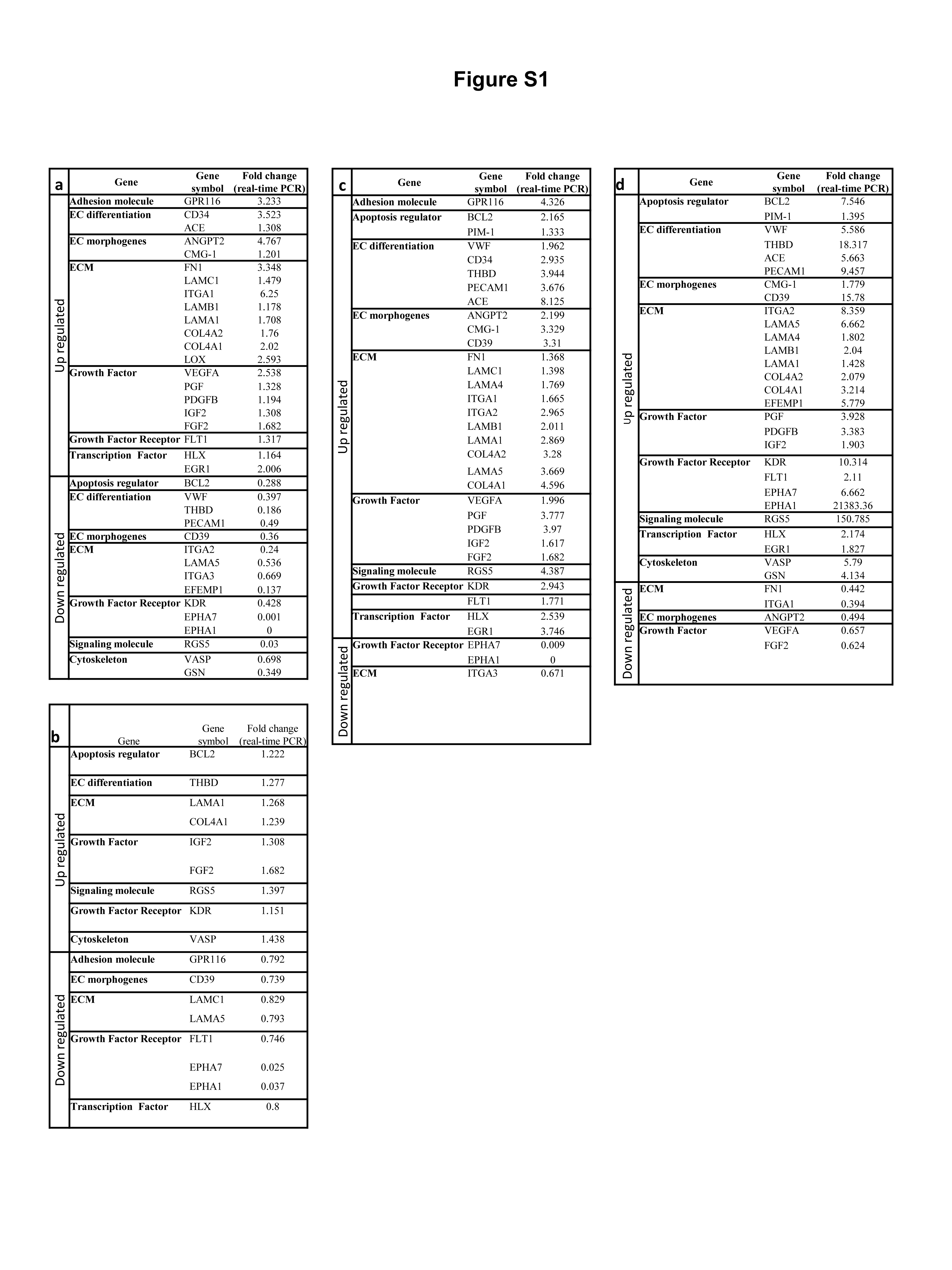

Supplement: Figure S1 — Expression profiles of EC morphogenesis-related genes. Quantitative real-time PCR analyses, using human specific primers were performed on islet-EC settings following 8 days of culture (a) with HFF in a 3D multicellular culture system relative to islet-EC cultured without HFF in a 3D co-culture (3D tri-culture vs. 3D co-culture) (b) with HFF in a 2D multicellular-culture relative to islet-EC cultured without HFF in a 2D co-culture system (2D tri-culture vs. 2D co-culture). (c) With HFF in a 3D multicellular culture relative to islet-EC cultured with HFF in a 2D multicellular culture system (3D tri-culture vs. 2D tri-culture). (d) Without HFF in a 3D co-culture relative to islet-EC cultured without HFF in a 2D co- culture system (3D co-culture vs. 2D co-culture). Values were normalized to human GAPDH. (TIF) [file pone.0040741.s001.tif]
