# Supplementary material for: Engineered Vascular Beds Provide Key Signals to Pancreatic Hormone-Producing Cells
Source: PLoS One. 2012 Jul 12;7(7):e40741. doi: 10.1371/journal.pone.0040741 (PMC3395696; doi:10.1371/journal.pone.0040741)
Supplement: Table S1 — List of primers used for gene expression analysis. (DOCX) [file pone.0040741.s002.docx]

**Table S1**

Primers list:

| Gene description | Gene symbol | Taqman Assay-on-Demand ^TM^ ID |
| --- | --- | --- |
| Glyceraldehyde 3-phosphate dehydrogenase | GAPDH | Hs99999905_m1 |
| 18[S](http://en.wikipedia.org/wiki/Svedberg) ribosomal RNA | 18S rRNA | Hs99999901_s1 |
| Insulin | Insulin1 | Mm01259683_g1 |
| glucagon | Glc | Mm00801714_m1 |
| pancreatic and duodenal homeobox 1 | Pdx1 | Mm00435565_m1 |
| NK6 homeobox 1 | Nkx6-1 | Mm00454962_m1 |
| Glucose transporter type 2 | Glut2 | Mm00446224_m1 |
| V-maf musculoaponeurotic fibrosarcoma oncogene homolog A | Mafa | Mm00845209_s1 |
| Proprotein convertase subtilisin/kexin type 1 | Pc1/3 | Mm00479023_m1 |
| Glucokinase | GCK | Mm00439129_m1 |
| Integrin-beta1 chain | Itgb1 | Mm01253227_m1 |
| vascular endothelial growth factor A | Vegfa | Mm01281447_m1 |
| CD34 | CD34 | Hs00156373_m1 |
| Angiopoietin-2 | ANGPT2 | Hs00237017_m1 |
| Angiotensin converting enzyme | ACE | Hs00174179_m1 |
| B-cell CLL/lymphoma 2 | BCL2 | Hs00236808_s1 |
| Collagen IV alpha1 chain | COL4A1 | Hs01007469_m1 |
| Collagen IV alpha2 chain | COL4A2 | Hs01098873_m1 |
| early growth response | EGR1 | Hs00152928_m1 |
| Ectonucleoside triphosphate diphosphohydrolase 1 | CD39 | Hs00169946_m1 |
| EGF-containing fibulin-like extracellular matrix protein 1 | EFEMP1 | Hs00244575_m1 |
| EPH receptor A7 | EPHA7 | Hs00177891_m1 |
| EPH receptor A1 | EPHA1 | Hs00178313_m1 |
| Fibroblasts growth factor 2 | FGF2 | Hs00266645_m1 |
| Fibronectin | FN1 | Hs00167309_m1 |
| fms-related tyrosine kinase 1 | FLT1 | Hs00176573_m1 |
| gelsolin | GSN | Hs00609276_m1 |
| GPR116 | GPR116 | Hs00391810_m1 |
| homeobox transcription factor | HLX | Hs00172035_m1 |
| Insulin-like growth factor 2 | IGF2 | Hs00277496_s1 |
| Integrin-alpha1 chain | ITGA1 | Hs00235030_m1 |
| Integrin-alpha2 chain | ITGA2 | Hs00158148_m1 |
| Integrin-alpha3 chain | ITGA3 | Hs00233707_m1 |
| Kinase Insert Domain-containing Receptor | KDR | Hs00176676_m1 |
| Laminin-alpha1 chain | LAMA1 | Hs00300550_m1 |
| Laminin-alpha4 chain | LAMA4 | Hs00158588_m1 |
| Laminin-alpha5 chain | LAMA5 | Hs00245699_m1 |
| Laminin-beta1 chain | LAMB1 | Hs00158620_m1 |
| Laminin-gamma1 chain | LAMC1 | Hs00370385_m1 |
| lysyl oxidase | LOX | Hs00184700_m1 |
| pim-1 oncogene | PIM-1 | Hs01065498_m1 |
| Placental Growth Factor | PGF | Hs01119262_m1 |
| Platelet-derived growth factor | PDGFB | Hs00234042_m1 |
| Platelet-endothelial cell adhesion molecule1 | PECAM1 | Hs00169777_m1 |
| regulator of G-protein signaling 5 | RGS5 | Hs00186212_m1 |
| Thrombomodulin | THBD | Hs00264920_s1 |
| vascular cell adhesion molecule 1 | CMG-1 | Hs00365486_m1 |
| Vascular endothelial growth factor | VEGFA | Hs00900054_m1 |
| Von Willebrand Factor | VWF | Hs00169795_m1 |
| Vasodilation-stimulated phosphoprotein | VASP | Hs00163444_m1 |
